# Construction Notes

**Published:** 1894-08-11

**Authors:** 


### CONSTRUCTION NOTES.

**Convalescent Home, Folkestone.**

The lease of the present Convalescent Home transpires this year. It is, therefore, proposed to erect a new building, which shall be thoroughly adapted to the purposes of a convalescent home. An admirable site has been secured on the East Cliff overlooking the sea. The plans, which are prepared by Mr. W. A. Hughes, architect, of 3, Dean's Yard, Westminster, show the proposed building to contain on the ground floor dining-room, matron's room, general sitting-room, women's sitting-room, men's sitting-room, kitchen, and other office accommodation; also men's and women's lavatories, well lighted hall, and vestibule. On the first floor there will be sleeping accommodation for twenty-six persons in as many well-lighted and ventilated cubicles; also visitors' bed-room, matron's bed-room, and additional bed-room for three beds. This floor will also contain two bath-rooms, two water-closets, and two linen closets. The men's and women's portions will be cut off entirely from each other. The front portion of the building has an additional floor, and will contain sleeping accommodation for thirteen persons, cistern-rooms, and store-rooms. It is proposed to divide the ground floor from the upper portion of the building by fire-proof flooring All the rooms and corridors will be well lighted, ventilated, and heated. All the external walls are to be built with a cavity between the inner and outer casings to ensure dryness, and the whole of the site underneath the ground floor will be covered with six inches of Portland cement concrete. An open porch, with verandahs on each side, will be provided on the ground floor, with balconies over. The total estimated cost of the undertaking is £3,250, including boundary walls and fences.

**Luton Children's Home.**

The form of this building, which has just been completed, is in the form of a large central block and two wings. The front entrance opens on to a spacious hall, in which broad stairs lead to the two stories. On the left of the entrance is the children's dining and board rooms. On the opposite side is the nurses' sitting-room. At one end of the first storey is the convalescent ward, for small inmates, where seven beds are situated. In an operating-room along the corridor is space for two beds, and close by is the sick ward, which contains accommodation for seven more children. On this floor also are the nurses' bed-rooms, while on the upper floor are situated an isolation ward with two cots and a chair bedstead, a nurse's room, and servants' bed-room. The fittings throughout the buildings are of the best possible description, and the home possesses complete provision in the shape of the linen closets and similar convenience. At the rear are found the usual offices. The architects are Messrs. J. R. Brown and Son. At present accommodation for twenty patients has been provided, and a start will be made with sixteen patients.

**Memorial Wing, Cookridge Convalescent Home.**

We announced some time ago that a memorial wing, dedicated and named after Edward Jackson, was to be added to the above institution, such extension to be called the Edward Jackson Memorial Wing. The premises of the home have for several years been unable to provide for all claims for admission, and it was considered desirable and necessary to provide further accommodation. This new wing, which has now been completed from suitable designs by Mr. Walter A. Hobson, architect, Leeds, harmonizes with the Gothic building to which it has been added. It is largely devoted to the sleeping accommodation of the home. On the ground floor, in addition to two dormitories, there is a new men's day-room, a conspicuous feature of which is a mural tablet, being an inscription in memory of the late benefactor of Leeds after whom their wing is named. The room opens by folding doors into what was formerly the men's day-room, but which has now been extended out, and will now serve as a dining-hall for the whole of the patients of the hospital. The new dining-room is so arranged as to afford communication between the original and the new building. Another communication is by an ornamental glass roof, which in unfavourable weather is used as a recreation-room, adjoining which are smoke, billiard, bath, and dressing rooms. The recreation-room adjoins the dining-hall, with which it runs parallel, and at its eastern end is the door leading into the new wing. On each side of the staircase hall, further along the corridor, are dormitories, one having accommodation for eleven beds and the other for eight. There are four other dormitories of similar design, two of which are situated on the first floor and two on the second floor. The upper rooms are reached by a central hall staircase, open from floor to roof, and by means of which the whole of the dormitory may be ventilated. Each domitory is efficiently supplied with fresh air. ' In addition to the two dormitories on the first floor is a third sleeping room for eleven patients. On each of the upper floors of the new wing there is a porter's room and lavatory. A new boiler, boiler-house, and chimney have been erected. The contractors for the general work are Messrs. Cross and Carter, of Leeds.

**New Union Infirmary at Dewsbury.**

The opening ceremony recently took place of the new pile of buildings which are to serve as an infirmary or hospital, and which are erected in close proximity to the workhouse at Staincliffe. Plans for these were accepted from Messrs. Holtom and Fox, of Dewsbury. The infirmary is planned on the pavilion principle, and has a frontage of 260 ft., and consists of the following buildings: Administrative block, two bed-rooms, two pavilions, and mortuary. The administrative block contains the doctors' sitting-rooms, the dispensary, committee-room, and two waiting-rooms on the ground floor. On the first floor is the lying-in ward, two labour wards, and a nurses' kitchen with pantry attached. On the second floor are five nurses' bed-rooms and a sitting-room for off-duty work. Each floor is fitted up with baths, lavatories, closets, &c. In the basement several large store-rooms are provided. The two pavilions are placed at a distance of 60 feet from the administrative block on the right and the other on the left, a connecting corridor being between. The pavilions are two storeys in height, each containing two large wards for 28 beds, two smaller wards for three beds, and two large day-rooms, two kitchens, with pantry, store-room, and linen cupboards, a wide staircase giving access to these rooms. At the extreme west end two small wards are provided for venereal cases, for four beds each, cut off from the other portion of the building and having a separate entrance and yard. Each of the above wards are fitted up with all necessary lavatories, &c. The buildings are constructed in a very substantial manner, the staircases being solid stone, and the corridors are made fireproof.

**Branch Home of the Bristol Hospital.**

The committee of the Bristol Hospital for Sick Women and Children have reconstructed and arranged a previous private home at Weston-super-Mare for the benefit of patients at the parent institution at Bristol. They have built a large play-room 38 ft. by 21 ft., which will be floored with wood blocks set in concrete, and will make a splendid play-room for the small inmates when they are unable to go out of doors. On the floor over this will be the matron's bed-room, a bath-room, and large linen press, and a new dormitory for children. The present dormitories are being considerably altered, and various outbuildings, such as larder, scullery, lavatory, &c., are constructed. The architect designing these alterations and extensions is Mr. John Pizey, of All Saints Court, Bristol, and the builder Mr. H. H. Forse, of Bristol and Weston-super-Mare.

**Cornelia Hospital, Poole.**

Some weeks past the Cornelia Hospital, Poole, has been closed that the building might be thoroughly renovated. Various improvements have been effected, and the hospital has been painted, cleaned, papered, &c., and an extra bath-room and other offices have been provided. Hot-water apparatus on the most improved system has been fixed throughout. The whole work, excepting the fixing of the hot-water, has been carried out by Mr. W. Wheeler, builder, of Longfleet. There are altogether seven wards, with 30 beds, and 15 of them are now occupied. Lady Wimborne, besides having the work of renovation carried out, has provided additional furniture for the wards, all of which presented a very cheerful and comfortable appearance.

